# Association of symptoms and interval breast cancers in the mammography-screening programme: population-based matched cohort study

**DOI:** 10.1038/s41416-018-0308-2

**Published:** 2018-11-07

**Authors:** Deependra Singh, Joonas Miettinen, Stephen Duffy, Nea Malila, Janne Pitkäniemi, Ahti Anttila

**Affiliations:** 1 Mass Screening Registry, Finnish Cancer Registry, FI-00130 Helsinki, Finland; 2 Epidemiology group, Department of Health Sciences, University of Tampere, FI-33520 Tampere, Finland; 3 Wolfson Institute of Preventive Medicine, Queen Mary University of London, Charterhouse Square, London, England

**Keywords:** Cancer epidemiology, Breast cancer, Signs and symptoms

## Abstract

**Background:**

We assessed the association between symptoms reported at breast cancer screening visits and interval cancers (ICs) in a prospective manner.

**Methods:**

This population-based matched cohort study uses data of the Finnish National Breast Cancer Screening Programme that invites women aged 50–69 years old during 1992–2012. Subjects who attended screening with symptoms were matched with asymptomatic reference cohorts based on age at screening visit, year of invitation, number of invited visits and municipality of invitation. The primary outcome was ICs.

**Results:**

Women with a lump had a threefold (hazard ratio 3.7, 95% confidence interval (CI) 3.0–4.6) risk of ICs and a higher risk (hazard ratio 1.7, 95% CI 1.4 to 2.0) at the subsequent visit compared with those without a lump. The fatal interval cancer risk increased by 0.39 per 1000 screens with a lump. The cumulative incidences of interval cancer increased within a month of a mammography-negative visit with a lump and after about 6 months of the visit with retraction or nipple discharge.

**Conclusion:**

Women with breast symptoms have a clearly increased risk of interval breast cancer after the screening visit. Our findings indicate the need for different screening strategies in symptomatic women.

## Background

Beyond the randomised trial environment, there is some uncertainty as to the underlying incidence of breast cancer and the rate of overdiagnosis in the screening population.^1,2^ Along with screening performance,^3–5^ rates and proportions of interval cancers (ICs) are important indicators for assessing the effectiveness and quality of screening.^2,6–8^ The substantial proportion (about a third) of incident breast cancer diagnosed outside the mammography-screening programme^9,10^ likely indicates that there is room for improvement in the detection capability of the mammography-screening programme. Earlier observational studies^7,11–15^ have highlighted several reasons for the increased proportion of ICs in the screening programme. However, in terms of equity within the screening population, it is reasonable to aim for similar interval cancer rates or at least similar proportions of cancers arising as interval cases for the various heterogeneous groups participating in the screening. Furthermore, options to modify screening policies should be considered for high-risk groups. A shorter screening interval may be justified, for instance, if the interval cancer rate is significantly high.

Based on the European Union (EU) guidelines, screening is meant for unselected target population.^7^ Earlier studies from Finland have indicated that a noticeable proportion (~ 2–3%) of women have clinically significant symptoms when they participate in breast cancer screening.^16,17^ Most but not all symptomatic women will have further assessments with ultrasound, additional mammograms or other methods; if these return negative results, the women return to the normal, biennial screening interval. Studies on breast symptoms (such as a lump, retraction or nipple discharge) indicate an increased risk of breast cancer^16–19^ at the cost of rise in false-positive findings. The relations of symptoms with interval breast cancers and screen-detected cancers (SDCs) at the subsequent visit have never been studied.

We investigated whether women reporting breast symptoms at screen are at a higher risk of developing subsequent breast cancers (ICs and cancers diagnosed at the next screen) than those without symptoms. To create foundations to modify the screening policies in high-risk groups, we estimated the cumulative incidence of ICs and fatal interval breast cancers, and compared the respective incidences in women with and without symptoms. The quality measures of screening mammography were compared between visits among subjects with and without symptoms to gather evidence for improving programme performance.

## Methods

### Study design, data source and study population

**Fig. 1:**
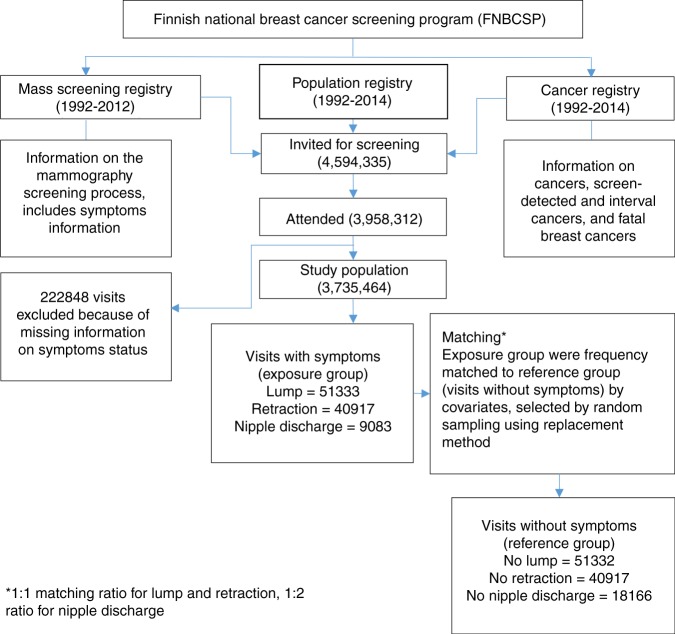
Flow diagram of study settings

Our matched cohort study design was based on the follow-up of the ongoing Finnish National Breast Cancer Screening Programme that began in 1987. Biennial screening visits made by women aged 50–69 years between 1991 and 2012 were selected. Three registries, the Finnish Cancer Registry (1953–2014), the Mass Screening Registry (1992–2012) and the Central Population Registry (1992–2014) were used to extract information on the study participants at the individual level. The Mass Screening Registry was used to extract information on demographic, symptomatic and screening procedure factors, including recalls and referral data that have been shown to be valid and of high quality. All individual visits were linked to the Finnish Cancer Registry database to retrieve information on breast cancers (screen-detected and ICs). This included histological findings and potential death from incident breast cancer. The Population Registry was used to identify possible dates of death or emigration, and where applicable, the cause of death was retrieved from Statistics Finland. Fig. 1 shows the flow diagram of the study design.

### Exposure group (screening visits with symptoms)

The exposed group is defined as visits by women with breast symptoms (lump, retraction and nipple discharge) reported at a given screening round. This group contained all visits with at least one symptom reported. Different symptoms (lump, retraction, nipple discharge) were analysed separately, which in technical terms assumes that the first occurrence of any symptom was independent given the covariates. For example, if more than one symptom was reported at a single visit then each symptom was analysed separately. Here, the index visit meant any screening visit with any given symptom.

### Reference group (screening visits without symptoms)

The reference group is defined as visits by women with no reported breast symptoms in the screening history before the index visit. The women from the reference group can later be the part of the exposed group if symptoms are reported at future screening visits. Thus, symptoms are time-dependent covariates. Individual sets of reference visits for each symptom—altogether three sets—were formed by matching.

### Matching

The three exposed groups were frequency matched to the reference groups by age at the screening visit (within 2 years), year of invitation (2-year band), number of visits in the past and municipality of invitation. Visits with symptoms were then aggregated based on matching variables (and other covariates). Each symptom stratum was matched to the viable controls (reference visits) by random sampling. Random controls were selected, based on the matching variables above, as many times as the number of visits in each stratum of symptoms by the replacement sampling method. Hence, a single control had the possibility to be randomly selected more than once to the same stratum. Based on our assumption of an effect size and required power of 0.80, the exposed-to-reference-visits ratio was 1:1 for lump and retraction, and 1:2 for nipple discharge.

### Outcome assessment

ICs were defined as breast cancers diagnosed in screened women before the next screening visit or within a period equal to a screening interval with (i) negative mammography at the index visit (i.e., test negative); (ii) positive mammography at the index visit, but negative further assessment (i.e., episode negative); and (iii) positive further assessment but a date of diagnosis > 6 months after mammography.^2^ SDCs were defined as primary breast cancer diagnosed among the screening attendees within 6 months following an abnormal mammogram (test positive). The subsequent round SDCs were analysed following the index visits with or without symptoms if the women attended the subsequent round. In addition, cancers were sub-grouped into in situ carcinomas and non-localised breast cancers. Fatal cancers were defined as those breast cancers that resulted in death during follow-up.

### Follow-up

The follow-up time started from the index visit in 1 January 1992 to 31 December 2012 and ended at the date of emigration or death, upon diagnosis of interval cancer or at the end of the follow-up—i.e., 31 December 2014—whichever occurred first. Cancer cases diagnosed among those screened up to 31 December 2012 and followed up to 31 December 2014 (for those screened in 2011 and 2012) were divided into ICs and subsequent SDCs using Finnish Cancer Registry data. Considering possible delays in the diagnosis date after positive mammography findings, a screening episode of 6-month intervals was used in the definition of detection mode. Thus, the follow-up time for ICs started at 7 months for episode negative visits and at 1 month for test negative visits and ended at the date of the subsequent screening visit at 23 months.

### Statistical analysis

We compared breast cancer risk and the risk of breast cancer death using Cox proportional hazard regression among women with and without reported symptoms at the index screen. Confidence intervals were computed exactly from parameter likelihoods. The analyses were adjusted for age at the screening visit. We calculated the incidence rate of interval cancer between the screens (from the index screen and the 6-month episode to the subsequent screening visit) separately for test negatives and episode negatives.

We also evaluated the programme characteristics using basic statistics. A test sensitivity was estimated as the number of visits with a positive mammography test and diagnosis of cancer at screen divided by the sum of SDCs plus ICs diagnosed after negative test results. Episode sensitivity was calculated as the number of visits with a diagnosis of cancer in a full diagnostic process in the screened population divided by all cancers detected in a screening round among attenders. Similarly, the positive predictive value (PPV) was assessed as the number of visits with a positive mammography test and diagnosis of cancer divided by the number of test positives. The negative predictive value (NPV) was estimated as the number of visits with a negative test result and no cancer diagnosed divided by the number of test negatives. All statistical analyses were performed using R-3.4.0.

## Results

Over the study period of 21 years, a lump was reported at 51,333 visits and retraction at 40,917 visits. These visits were matched to an equal number of asymptomatic visits. There were 9083 visits with nipple discharge, and they were matched with double the number of reference visits (i.e., 18,166 visits) without nipple discharge. Detailed numbers of the potentially eligible and the confirmed eligible population included in the study are shown in Fig. 1.

The mean age at a screening visit did not differ between visits with and without symptoms (mean age 55.7 vs 56.1 years for visits with and without a lump, respectively). Table 1 shows the characteristics of the final study cohort. About one in three women who reported a lump or nipple discharge and one in eight women who reported retraction were first-time attendees. The first and subsequent attendee’s proportions were similar between visits with and without symptoms. More than 80% of ICs and the subsequent round’s SDCs, irrespective of reported symptoms status, were not recalled for further assessment at the index visit.

**Table 1:** Cohort characteristics

Characteristics		Lump (*n* = 51,333)		Retraction (*n* = 40,917)		Nipple discharge*	
		Yes (%)	No (%)	Yes (%)	No (%)	Yes (%) (*n* = 9083)	No (%) (*n* = 18,166)
Age at index visit (mean, SD)		55.7 (4.6)	56.1 (4.5)	57.7 (4.9)	56.6 (4.8)	54.9 (4.6)	56.1 (4.7)
Attendance	First attendance	18,305 (35)	17,954 (35)	5012 (12)	4959 (12)	2904 (32)	5643 (31)
	Subsequent attendance	33,028 (64)	33,379 (65)	35,905 (87)	35,958 (87)	6179 (68)	12,523 (68)
Previous round attendance	Yes	31,571 (61)	32,228 (62)	34,168 (83)	34,263 (83)	5842 (64)	12,069 (66)
	No	19,762 (38)	19,105 (37)	6749 (16)	6654 (16)	3241 (35)	6097 (33)
Period of visit	1992–1997	15,478 (30)	15,520 (30)	0	0	1134 (12)	2298 (12)
	1998–2002	7535 (14)	7493 (14)	3552 (8.7)	3552 (8.7)	1673 (18)	3316 (18)
	2003–2007	11,791 (23)	11,804 (23)	12,996 (31)	13,190 (32)	2872 (31)	5671 (31)
	2008–2012	16529 (32)	16,516 (32)	24,369 (59)	24,175 (59)	3404 (37)	6881 (37)
Recall (test positives)							
Invasive cancers	Screen-detected cancers	1440 (2.8)	174 (0.34)	461 (1.1)	192 (0.47)	66 (0.73)	83 (0.46)
	Interval cancers	70 (0.14)	7 (0.01)	16 (0.04)	5 (0.01)	12 (0.13)	6 (0.03)
	Subsequent screen at next round	34 (0.07)	6 (0.01)	11 (0.03)	7 (0.02)	4 (0.04)	3 (0.01)
Fatal breast cancers	Screen-detected cancers	215 (0.41)	11 (0.02)	38 (0.09)	6 (0.01)	4 (0.04)	2 (0.01)
	Interval cancers	8 (0.01)	2 (0.01)	2	2	0	0
	Subsequent screen at next round	1 (0.01)	0	1 (0.01)	0	0	0
No recall (test negatives)							
Invasive cancers	Screen-detected cancers	0	0	0	0	0	0
	Interval cancers	317 (0.62)	96 (0.19)	138 (0.34)	96 (0.23)	40 (0.44)	35 (0.19)
	Subsequent screen at next round	230 (0.45)	149 (0.29)	144 (0.35)	135 (0.33)	28 (0.31)	57 (0.31)
Fatal breast cancers	Screen-detected cancers	0	0	0	0	0	0
	Interval cancers	32 (0.06)	18 (0.03)	13 (0.03)	5 (0.01)	5 (0.05)	2 (0.01)
	Subsequent screen at next round	18 (0.03)	7 (0.01)	4 (0.01)	4 (0.01)	0	1 (0.01)

Values are numbers (percentage) unless stated otherwise

*For each visits with nipple discharge were matched with two visits without nipple discharge

Both the test and episode sensitivity of the mammography was higher for visits with a lump or retraction compared with those without such symptoms (82 and 75% vs 64 and 63% for a lump vs no lump; 77 and 76% vs 67 and 66% for retraction vs no retraction; Table 2). Likewise, the PPV of mammography was higher for retraction and a lump, compared with those without these symptoms. However, the specificity of mammography was clearly lower for visits with a lump (88%) than those without (98%). Some decrease in specificity was also seen for retraction and nipple discharge.

**Table 2:** Performance quality measures of screening mammography in relation to symptoms status

Symptoms		Screening episode	Mammography test			
		Sensitivity % (95% CI)	Sensitivity % (95% CI)	PPV % (95% CI)	NPV % (95% CI)	Specificity % (95%CI)
Lump	Yes	74 (71–78)	81 (80–83)	19 (19–20)	99 (99–99)	88 (88–88)
	No	62 (56–68)	64 (58–70)	12 (11–13)	99 (99–99)	97 (97–97)
Retraction	Yes	75 (72–79)	76 (73–80)	28 (26–29)	99 (99–99)	97 (96–97)
	No	65 (59–70)	66 (60–72)	17 (16–19)	99 (99–99)	97 (97–98)
Nipple discharge	Yes	55 (46–65)	62 (52–71)	8.1 (6.9–9.4)	99 (99–99)	91 (91–92)
	No	66 (57–75)	70 (61–78)	15 (13–17)	99 (99–99)	97 (97–97)

*PPV* positive predictive value, *NPV* negative predictive value, *CI* confidence interval

### Incidence of screen-detected and ICs, subsequent SDCs and fatal ICs

In total, 1440 (2.8%) SDCs and 387 (0.7%) ICs (ICs) were diagnosed in those who reported a lump compared with 174 (0.3%) SDCs and 103 (0.2%) ICs in those without a lump, respectively (Table 3). The proportions of SDCs and ICs were higher also for retraction and nipple discharge compared with those without these symptoms. The age-adjusted risk of SDCs was significantly higher in those who reported a lump (adjusted hazard ratio 8.2, 95% CI 7.0–9.7), retraction (adjusted hazard ratio 2.3, 95% CI 2.0–2.8) or nipple discharge (adjusted hazard ratio 1.5, 95% CI 1.1–2.3) compared with those without symptoms. In addition, the age-adjusted risk of ICs was significantly higher for a lump (adjusted hazard ratio 3.7, 95% CI 3.0–4.6), retraction (adjusted hazard ratio 1.5, 95% CI 1.1–1.9), and nipple discharge (adjusted hazard ratio 2.4, 95% CI 1.6–3.7) compared with those without these symptoms. The risk of SDCs in the subsequent round was significantly higher only after visits with a lump compared with those without a lump (adjusted hazard ratio 1.6, 95% CI 1.3–2.0). The risk of in situ interval carcinomas or subsequent screen-detected carcinomas was also higher in visits with a lump and nipple discharge compared with a visit with no symptoms. The risk of non-localised interval breast cancer as well as SDCs in the subsequent round were also greater for all three symptoms in comparison with visits without symptoms.

**Table 3:** Frequency (per 1000 screening visits) and age-adjusted risk of cancer outcomes in those who reported symptoms compared with those without symptoms at screen

Outcomes	Lump (*n* = 51,333)			Retraction (*n* = 40,917)			Nipple discharge*		
	Yes (per 1000)	No (per 1000)	Age-adjusted hazard ratio (95% CI) with reference to no lump	Yes (per 1000)	No (per 1000)	Age-adjusted hazard ratio (95% CI) with reference to no retraction	Yes (per 1000) (*n* = 9083)	No (per 1000) (*n* = 18,166)	Age-adjusted hazard ratio (95% CI) with reference to no nipple discharge
Invasive cancers									
Screen-detected cancers	1440 (28)	174 (3.4)	8.3 (7.1–9.7)	461 (11)	192 (4.7)	2.4 (2–2.8)	66 (7.3)	83 (4.6)	1.7 (1.2–2.3)
Interval cancers	387 (7.5)	103 (2.0)	3.8 (3–4.7)	154 (3.8)	101 (2.5)	1.5 (1.2–1.9)	52 (5.7)	41 (2.3)	2.5 (1.7–3.8)
Subsequent screen at next round	264 (5.1)	157 (3.1)	1.7 (1.4–2.1)	156 (3.8)	158 (3.9)	1.1 (0.87–1.4)	32 (3.5)	72 (3.9)	1.0 (0.64–1.5)
In situ carcinomas									
Screen-detected cancers	61 (1.2)	38 (0.74)	1.6 (1.1–2.4)	35 (0.86)	31 (0.76)	1.1 (0.70–1.8)	15 (1.7)	16 (0.88)	1.9 (0.92–3.8)
Interval cancers	35 (0.68)	9 (0.18)	3.9 (1.9–8.6)	7 (0.17)	6 (0.15)	1.2 (0.39–3.6)	9 (0.99)	4 (0.22)	4.0 (1.3–14)
Subsequent screen at next round	159 (3.1)	102 (1.9)	1.5 (1.2–1.9)	76 (1.9)	66 (1.6)	1.2 (0.83–1.6)	32 (3.5)	32 (1.8)	2.0 (1.2–3.3)
Non-localised cancers									
Screen-detected cancers	693 (13)	57 (1.1)	12 (9.2–16.0)	230 (5.6)	61 (1.5)	3.5 (2.6–4.7)	25 (2.8)	27 (1.5)	1.9 (1.1–3.2)
Interval cancers	178 (3.5)	57 (1.1)	3.4 (2.5–4.6)	79 (1.9)	48 (1.2)	1.7 (1.2–2.4)	20 (2.2)	18 (0.99)	2.2 (1.2–4.2)
Subsequent screen at next round	649 (12)	438 (8.5)	1.5 (1.3–1.7)	244 (5.9)	182 (4.5)	1.4 (1.1–1.7)	110 (12)	181 (9.9)	1.2 (0.96–1.6)
Fatal cancers									
Screen-detected cancers	215 (4.2)	11 (0.21)	19 (11–38)	38 (0.93)	6 (0.15)	6.3 (2.9–16)	4 (0.44)	2 (0.11)	4.0 (0.78–28)
Interval cancers	40 (0.78)	20 (0.39)	2 (1.2–3.5)	15 (0.36)	7 (0.17)	2.1 (0.90–5.6)	5 (0.55)	2 (0.11)	5.0 (1.1–34)
Subsequent screen at next round	19 (0.37)	7 (0.14)	2.7 (1.7–4.1)	5 (0.12)	4 (0.07)	1.3 (0.45–2.7)	0	1 (0.05)	NA

*For each visits with nipple discharge were matched with two visits without nipple discharge; *CI* confidence interval, *NA* not available

The age-adjusted risk of dying from breast cancer was significantly higher in those who reported a lump and were diagnosed with invasive cancers (SDCs = adjusted hazard ratio 19, 95% CI 11–38; ICs = adjusted hazard ratio 2.0, 95% CI 1.1–3.4; subsequent round cancers = adjusted hazard ratio 2.7, 95% CI 1.6–4.1) compared with those without a lump (Table 3). In addition, the risk of dying was higher in those who reported retraction and were diagnosed with SDCs (adjusted hazard ratio 6.3, 95% CI 2.8–16) compared with those without retraction. Only a few deaths occurred during the follow-up in those who reported nipple discharge.

### Cumulative incidence of breast cancers during the screening interval

**Fig. 2:**
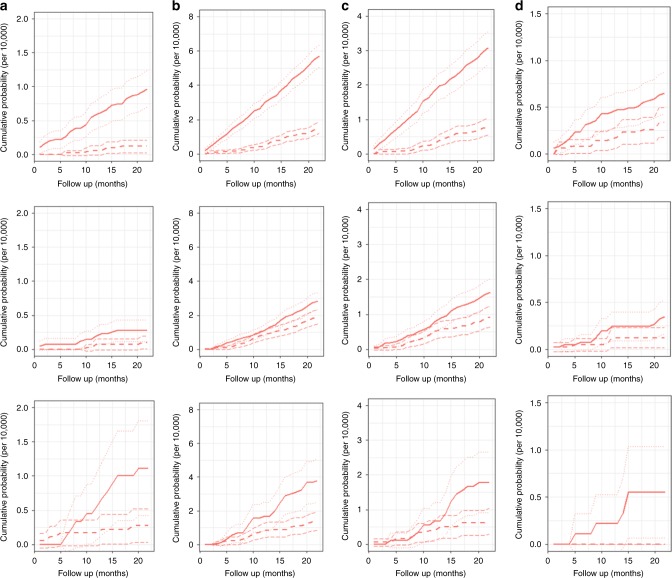
**a–d** Cumulative incidence of invasive (per 1000): **a** recalled ICs; **b** not recalled ICs; **c** non-localised ICs; **d** fatal interval cancers. Note: the confidence intervals lines for cumulative incidence are indicated by light dotted lines in symptomatic and asymptomatic groups

The incidence of ICs after a visit with a lump or nipple discharge increased rather rapidly after the visit in all the studied progression types (Fig. 2 a–d). When using the cumulative incidence of the asymptomatic over the whole interval as a reference, the same level of ICs was reached in only about six months after visits with a lump and 12 months after visits with nipple discharge. Remarkably, in mammography-negative visits with a lump, the cumulative incidence curve detached from the no lump curve immediately after the first month of visit, whereas such a difference was not observed for the other symptom types.

## Discussion

In this population-based study with 21 years of screening (the follow-up is restricted to 23 months, although the study period was 21 years), we observed strong associations between symptoms and breast cancer risks. Women reporting a lump at a screening visit had a threefold risk of ICs compared with those with no symptoms, also including subsequent SDCs. The (cumulative) incidence of interval cancer was higher in those who reported a lump irrespective of the mammography findings (test negatives or episode negatives) compared with those without symptoms at the index visit. Likewise, retraction and nipple discharge were significantly associated with increased risks of interval breast cancers. In absolute terms, per 1000 women who attended and reported a lump, seven women were diagnosed with invasive interval breast cancers, i.e., within 24 months compared with about two cancers diagnosed without symptoms. The fatal interval cancer risk increased by 0.39 per 1000 screens with a lump.

Women with symptoms had a clearly increased ‘background’ risk of breast cancer; the conventional screening performance measures—such as sensitivity, PPV and specificity—did not fully assess this aspect. The cumulative incidence patterns as well as the detection of cancers during the subsequent round provided direct evidence of the need for risk-adjusted screening and a better diagnostic work-up in the symptomatic women. The diagnostic work-up could include developing better reading and recall criteria in screening mammograms, a more detailed indication for biopsies in the further assessment, and early recall for those with high-risk symptoms for whom the screening test or further assessment proves negative.

### Strengths and limitations of the study

To our knowledge, this is the first population-based study to analyse the association between symptoms and interval and fatal breast cancers. Our study has several strengths. First, we included all screening visits with symptoms from the start of the mammography-screening programme (over 21 years) and compared them with visits without symptoms. Since this was a population-based service-screening programme, the selection bias was minimal. Second, we could use validated prospectively collected register data with no recall bias.^20^ Validation of the cancer diagnosis (screen-detected and ICs) and death from cancer using national and covering data sources (Finnish Cancer Registry and Statistics Finland) and the use of unique personal identifier for individual-level linkage eliminates the possibility of selective misclassification. Third, we matched the symptomatic visits to asymptomatic visits by possible confounding baseline characteristics to minimise bias in the risk estimates. As a result, we found no significant difference in the background variables between visits with and without symptoms. Finally, the programme process and outcome indicator definitions used in this study adhere to those defined by WHO-IARC and mentioned in EU guidelines on breast cancer screening and diagnosis,^7,21^ including the provision of relevant information describing the performance and also failures during the various steps of the screening process.

Our study also has potential limitations. The symptom information was based on the women’s reporting in the past 2 or 6 months and a check by a radiographer at the visit. The radiographer’s inspection is likely to be less comprehensive than a full clinical examination. The collection of symptom information is mainly done in order to support the interpretation of the mammograms. However, in almost every case—if not all—the radiographer or nurse examines the breast to confirm the presence of symptoms (mainly a lump and retraction) before the mammography is performed. Thus, there are reasons to consider the collected symptom information to be valid, albeit not perfect. A second potential limitation is that our estimates could have been confounded, because symptomatic women are more likely to attend than asymptomatic women. However, because of the high attendance rate in the Finnish mammography-screening programme (84% among the invited, the highest among any existing mammography-screening programme), and as 97% of attendees are asymptomatic, the attendance bias caused by symptoms is likely to be small. Third, we did not use the background incidence of breast cancer to estimate the interval cancer rate in the absence of screening, but instead used a detection method^22^ that takes into account cancers from the screening programme; thus, estimates are sensitive to overdiagnosis and lead time bias. Given the high coverage and attendance rate in the Finnish screening programme, it was not possible to find a comparable non-screened group to estimate the background incidence of breast cancer. In addition, there was no possibility to estimate background incidence of breast cancer in women with symptoms. We did not find any difference in the PPV of mammography, with and without symptoms, between the first and subsequent screening rounds (not shown in results); thus, the lead time bias because of prevalent screens is negligible. Furthermore, we used invasive breast cancers—and also advanced and fatal breast cancers, which would be less affected by overdiagnosis—to estimate the incidence rates and hazard ratios. Only 5% of all carcinomas in those with symptoms were in situ carcinomas.

Previous studies on the association between symptoms and the risk of breast cancer are limited because of factors such as study design, size, follow-up time and assessment of the possible outcome measures of breast cancer. Nonetheless, a few studies have found that the presence of a palpable lump is associated with a higher risk of SDCs.^18,19^ We are not aware of any studies that have assessed the relationship of symptoms with other outcome measures.

### Clinical and public health implications

Both the screening test and episode sensitivities tended to be higher in symptomatic women compared with the asymptomatic. Correspondingly, the screening specificity was lower in the symptomatic women. Of note, particularly in women with a lump, were the (episode) sensitivity losses in the further assessment during the index visits. Moreover, higher interval cancer incidence within six months after a negative mammography with a lump is a clear concern for the programme. This indicates that further assessment is needed more frequently (albeit with a potential loss of specificity), and there needs to be highly stringent diagnostic evidence for a decision not to carry out a full further assessment including a core biopsy in these cases. As most visits (~ 97%) were asymptomatic, the number of additional services would be rather small as well as the improvement of programme’s overall performance and outcome. But still, high-quality and clinically appropriate services are important for women having symptoms at a screening visit. One option is to recall all women with symptoms even if the mammography result is negative, as practised in Norway (< 0.3% of all those screened were recalled with symptoms).^23^ Doing this in Finnish programme would significantly lower the PPV of recall, as 2.5% of all screens had symptoms and only ~ 1 out of 10 symptomatic visits have been recalled.^17^

Taking into account the high incidence of advanced and fatal interval breast cancers in symptomatic women, it is likely that protection by biennial screening visit would clearly not be sufficient even after potential improvements in further assessments. We are not aware of recommendations for surveillance or follow-up of symptomatic women in the programme. Hence, we recommend a shorter screening interval for the symptomatic group so that the cumulative incidences of interval and fatal interval cancer would possibly become more equitable. For two of the studied symptoms (lump and nipple discharge), the interval cancer incidence increased so rapidly that the first follow-up visit could take place very shortly after the index visit. Finally, taking into account the probability of fatal screen-detected breast cancer is higher in women with a symptom already at the index visit, women need to be better informed about symptoms and made aware that if a symptom occurs, it is not a good idea to wait until the next invitation to the programme. Guidelines for further assessment in patients presenting with symptoms before having a scheduled invitation according to the programme—as developed by National Health Service in the UK^24^—could be useful also in other settings.

## Concluding remarks

Women with breast symptoms at visits within the population-based breast cancer screening programme have a clearly increased risk of breast cancer. The cumulative incidence of invasive, advanced and fatal breast cancers, as well as the detection of them at the next screen, provide direct evidence for the need for risk-adjusted screening in symptomatic women—e.g., tailoring the management procedures at index visits and shortening the screening intervals for these women. This study provides clear evidence to update and support the EU guidelines^7^ recommendation that ensures sufficient attention being paid to symptomatic details provided by women. Our findings therefore have important implications for screening-aged women, radiologists, nurses and mammography-screening programmes overall.
