# Correction: Association of symptoms and interval breast cancers in the mammography-screening programme: population-based matched cohort study

**DOI:** 10.1038/s41416-019-0417-6

**Published:** 2019-03-06

**Authors:** Deependra Singh, Joonas Miettinen, Stephen Duffy, Nea Malila, Janne Pitkäniemi, Ahti Anttila

**Affiliations:** 1 Mass Screening Registry, Finnish Cancer Registry, FI-00130 Helsinki, Finland; 2 Epidemiology group, Department of Health Sciences, University of Tampere, FI-33520 Tampere, Finland; 3 Wolfson Institute of Preventive Medicine, Queen Mary University of London, Charterhouse Square, London, England

**Keywords:** Cancer epidemiology, Breast cancer, Signs and symptoms

**Correction to:** *British Journal of Cancer* (2018) **119**, 1428–1435; 10.1038/s41416-018-0308-2; www.bjcancer.com; published online 7 November 2018.

The authors report that the labels indicating the symptom types and no symptom lines in the original version of Fig. 2 were missing. The correct version of Fig. 2 with the labels included is provided below.

**Fig. 2:**
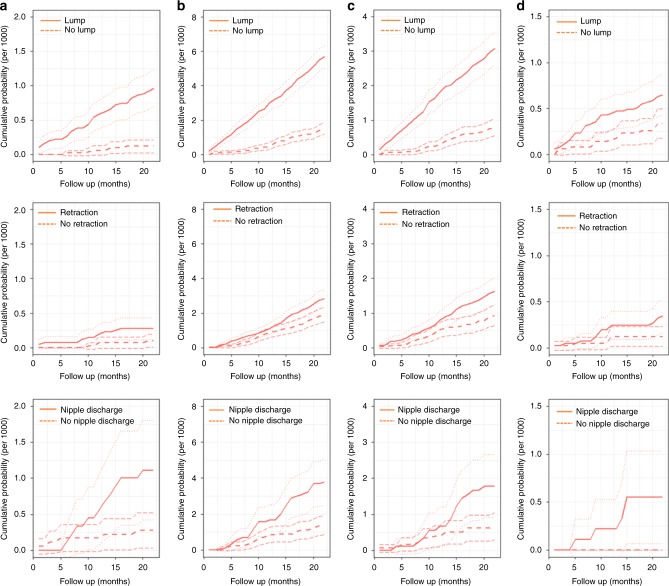
**a**–**d** Cumulative incidence of invasive (per 1000): **a** recalled ICs; **b** not recalled ICs; **c** non-localised ICs; **d** fatal interval cancers. Note: the confidence intervals lines for cumulative incidence are indicated by light dotted lines in symptomatic and asymptomatic groups

